# Oral Chinese patent medicines for acute myocardial infarction after percutaneous coronary intervention: A protocol for systematic review and network meta-analysis

**DOI:** 10.1097/MD.0000000000031927

**Published:** 2022-12-02

**Authors:** Wei Zhao, Fan-Jie Xiong, Shu-Gui Feng, Yan-Ming Li, Xing-Hua Lei, Shi-Jian Jia

**Affiliations:** a Xindu Hospital of Traditional Chinese Medicine Affiliated to Chengdu Medical College, Chengdu, China; b Luzhou Traditional Chinese Medicine Hospital Affiliated to Southwest Medical University, Luzhou, China.

**Keywords:** acute myocardial infarction, network meta-analysis, oral Chinese patent medicine, PCI, systematic review

## Abstract

Acute myocardial infarction (AMI) is a serious and fatal heart disease with one of the highest mortality rates in the world. In some countries, percutaneous coronary intervention (PCI) is the preferred reperfusion strategy after AMI, but it cannot achieve safe and effective treatment of AMI after PCI remains a challenging clinical problem. The potential of oral Chinese patent medicines to treat AMI after PCI has been demonstrated, but which type of oral Chinese patent medicines may be preferred remains controversial. The aim of this network meta-analysis was to investigate the efficacy and safety of multiple oral Chinese patent medicines in the treatment of AMI after PCI.

**Methods:**

We will conduct a literature search from China National Knowledge Infrastructure, formerly Chinese Biomedical Database (SinoMed), Wanfang Data, Chongqing VIP, PubMed, Embase, Web of Science and Cochrane Library (The Cochrane Database of Systematic Reviews) from their inception until to November 1, 2022, with language restricted to Chinese and English. Then, the study selection process will follow the Preferred Reporting Items for Meta-Analyses guideline, and the quality assessment will be conducted with Cochrane Collaboration’s tool. Pairwise and network meta-analysis will be conducted using the WinBUGS V.1.4.3.37 and STATA V.13. Additionally, sensitivity analysis, subgroup analysis, quality assessment, Small-study effects and publication bias will be performed.

**Ethics and dissemination:**

This work is based on published research and therefore does not require ethical approval. This review will be published in peer-reviewed journals.

**PROSPERO registration number:**

CRD42020188065.

## 1. Introduction

Acute myocardial infarction (AMI) is caused by acute coronary artery occlusion and blood flow interruption, resulting in continuous ischemia and hypoxia of myocardial cells. It is a serious fatal heart disease,^[1]^ one of the diseases with the highest mortality in the world, and a serious threat to human health and quality of life.^[2]^ According to its pathophysiology, clinic and prognosis, it is usually divided into 5 types.^[3]^ Generally speaking, coronary lumen stenosis is between 50% and 75%, and no symptoms of myocardial ischemia will occur in the quiet state. However, when the mood swings or under the stress state, hemodynamics will change,^[4,5]^ the atheromatous plaque ruptured, platelet aggregation formed thrombosis,^[6,7]^ and blood flow decreased or interrupted, resulting in myocardial infarction.^[8]^ In recent years, due to the gradual improvement of secondary prevention of coronary heart disease,^[9]^ the incidence and mortality of AMI have been decreasing.^[10,11]^ However, appear easily AMI acute or subacute left ventricular free wall rupture, ventricular septal rupture, acute mitral insufficiency, right ventricular infarction, wall of left ventricular aneurysm formation, and complications such as heart failure,^[12,13]^ and myocardial ischemia can seriously damage the systolic and diastolic function of the heart,^[14]^ from fear and anxiety can excite sympathetic nerve, increase cardiac load, further exacerbate the disease.^[15]^ Therefore, AMI should be treated immediately after onset, and actively prevent the occurrence of serious complications.

Recent studies have shown that the main mechanism of AMI initiation is plaque rupture or erosion accompanied by overburden thrombosis.^[16]^ This process is mainly related to inflammatory response, vascular endothelial injury, platelet activation and aggregation and other factors.^[17–20]^

Various clinical guidelines recommend a variety of treatment plans and drugs, and AMI should be mainly managed according to ALS algorithm (ABCDE).^[21]^ Management of myocardial infarction focuses on hemodynamic stability, pain relief, reduction of myocardial oxygen consumption, increase of myocardial oxygen supply and initiation of antithrombotic therapy.^[22]^ In countries with well-established percutaneous coronary intervention (PCI) networks and fast transmission times, PCI is the preferred reperfusion strategy. The European Society of Cardiology recommends the use of dual antiplatelet therapy after PCI, in which patients should take clopidogrel and aspirin for 9 to 12 months to reduce the incidence of major adverse cardiovascular events.^[23]^ However, if the drug dose is maintained for a long time, there will still be 10% to 15% patients with myocardial infarction, stroke and death.^[24,25]^ The incidence of “non-reflow” caused by reperfusion injury^[26]^ ranges from 5% to 50%,^[27,28]^ which may increase the size of myocardial infarction and affect the recovery of cardiac function, thus increasing the long-term end point events, leading to a large number of patients who cannot obtain satisfactory treatment response.^[29]^ Therefore, although PCI can clear the stenosis of the obstructive heart and blood vessels and improve the symptoms of angina pectoris, it cannot change the etiology of coronary heart disease, and achieving safe and effective treatment of AMI after PCI remains a challenging clinical problem.

As one of the characteristics of traditional Chinese culture, traditional Chinese medicine (TCM) has emerged as early as 3000 years ago and has rich therapeutic experience.^[30]^ Due to the lack of objective and quantitative evaluation criteria, TCM is regarded as complementary or alternative medicine in most western countries. Nowadays, TCM is becoming more and more popular in many developed and developing countries.^[31]^ A study has shown that TCM can be used as a supplementary and alternative method for primary and secondary prevention of cardiovascular diseases.^[32]^ Chinese patent medicine is an important part of TCM. Oral Chinese patent medicine is a general term of oral liquid dosage form (solution dosage form, suspension dosage form, emulsion dosage form) and oral solid dosage form (powder, capsule, tablet, pill).^[33]^ At present, oral Chinese patent medicine has been widely used in the clinical treatment of AMI after PCI in East and Southeast Asia.^[34,35]^

At present, there have been systematic reviews on the effectiveness and safety of single oral Chinese patent medicine in the treatment of AMI after PCI.^[36,37]^ However, the increasing variety of oral Chinese patent medicines means that the comparison between the 2 oral Chinese patent medicines is more complicated. For patients with AMI after PCI, there is no relevant research to confirm whether different oral Chinese patent medicines have different efficacy, as well as which oral Chinese patent medicines have the best efficacy and safety. Therefore, it is also a challenge for clinicians to choose which oral Chinese patent medicine to use as the first-line treatment. Online meta-analysis can be used to directly and indirectly compare the efficacy and safety of different oral Chinese patent medicines in the treatment of AMI after PCI, and to rank them.

Therefore, we decided to conduct a network meta-analysis to explore the relative effectiveness and safety of different oral Chinese patent medicines, and to provide evidence for their clinical application and precise positioning.

## 2. Objective

The objective of this systematic review and network meta-analysis is to compare the efficacy and safety of oral Chinese patent medicines for the treatment of AMI after PCI in the national essential medicines list of China.

## 3. Methods and analysis

The network meta-analysis protocol has been registered on the International Prospective Register of Systematic Review, the registration number is CRD 42020188065. Our protocol follows the Preferred Reporting Items for Systematic Reviews and Meta-Analysis Protocols guideline.^[38]^

### 3.1. Patient and public involvement

The network meta-analysis is a review and summary of previous research results, and therefore do not require the participation of patients and the public.

### 3.2. Eligibility criteria

#### 3.2.1. Study types.

We will include all relevant randomized controlled trials (RCTs), quasi-RCTs will be excluded, such as using in clinic order allocation of RCT, case report is not included.

#### 3.2.2. Participants.

Patients (age ≥ 18 years) who were diagnosed with AMI according to any standard criteria (such as Heart Disease and Stroke Statis-2020 Update: A Report From the American Heart Association) and who received PCI were included.^[39]^ Patients with serious fatal diseases would be excluded such as cancer.

#### 3.2.3. Interventions.

Any oral Chinese patent medicine used to treat AMI after PCI can be interfered. Regardless of dosage, dosage form, course of treatment and manufacturer, and these proprietary Chinese medicines are used in combination with conventional treatment. The experimental group was treated with oral Chinese patent medicine combined routine, while the control group was treated with oral Chinese patent medicine combined routine treatment or simple routine treatment, and the routine treatment measures should be the same between different RCT. Thus eligible combination: oral Chinese patent medicine + routine treatment versus routine treatment and oral Chinese patent medicine + routine treatment versus another oral Chinese patent medicine + routine treatment.

#### 3.2.4. Primary outcomes.

Primary outcomes included the rate of cardiovascular events and mortality.

1. Cardiovascular events: including recurrent angina pectoris, malignant arrhythmia, recurrent nonfatal myocardial infarction and heart source sex shock (during the observation time for medication).
2. Mortality: the mean follow-up time was no less than 6 months.

#### 3.2.5. Secondary outcomes.

1. Symptom improvement (including chest distress, shortness of breath).
2. Left ventricular ejection fraction improvement.
3. Duration of angina.
4. Cessation and reduction of nitroglycerin.
5. Adverse effects including bleeding events, toxic reactions, drug withdrawal, digestive symptoms, headache and dizziness.

### 3.3. Search strategy

A systematic search of the Chinese databases China National Knowledge Infrastructure, formerly Chinese Biomedical Database (SinoMed), Wanfang Data and Chongqing VIP Database for Chinese Technical Periodicals, as well as the PubMed, Embase, Web of Science and Cochrane Library (The Cochrane Database of Systematic Reviews) databases, from their inception to November 1, 2022. There will be no restrictions on language, country, publication status or year of publication. Take PubMed as an example, the detailed search strategy was shown in Table 1.

**Table 1:** Search strategy for PubMed.

Number	Search terms
#1	Acute Myocardial Infarction [Mesh]
#2	Myocardial infarction [Title/Abstract] OR Infarction, Myocardial [Title/Abstract] OR Infarctions, Myocardial [Title/Abstract] OR Cardiovascular Stroke [Title/Abstract] OR Cardiovascular Strokes [Title/Abstract] OR Stroke, Cardiovascular [Title/Abstract] OR Myocardial Infarct [Title/Abstract] OR Infarct, Myocardial [Title/Abstract] OR Myocardial Infarcts [Title/Abstract] OR Heart Attack [Title/Abstract] OR Heart Attacks [Title/Abstract]
#3	#1 OR #2
#4	Percutaneous Coronary Intervention [Mesh]
#5	Coronary Intervention, Percutaneous [Title/Abstract] OR Coronary Interventions, Percutaneous [Title/Abstract] OR Intervention, Percutaneous Coronary [Title/Abstract] OR Interventions, Percutaneous Coronary [Title/Abstract] OR Percutaneous Coronary Interventions [Title/Abstract] OR Percutaneous Coronary Revascularization [Title/Abstract] OR Coronary Revascularization, Percutaneous [Title/Abstract] OR Coronary Revascularizations, Percutaneous [Title/Abstract] OR Percutaneous Coronary Revascularizations [Title/Abstract] OR Revascularization, Percutaneous Coronary [Title/Abstract] OR Revascularizations, Percutaneous Coronary [Title/Abstract]
#6	#4 OR #5
#7	Oral Chinese Patent Medicines [Title/Abstract]
#8	musk baoxin pill [Title/Abstract] OR compound danshen dropping pill [Title/Abstract] OR qishen yiqi dropping pill [Title/Abstract] OR tongxinluo capsule [Title/Abstract] OR xinyue capsule [Title/Abstract] OR qiliqiangxin capsule [Title/Abstract]
#9	#7 OR #8
#10	randomized controlled trial[Publication Type]
#11	controlled clinical trial[Publication Type]
#12	randomized[Title/Abstract]
#13	randomly[Title/Abstract]
#14	#10 OR #11 OR #12 OR #13
#15	#3 AND #6 AND #9 AND #14

### 3.4. Study selection process

**Figure 1.**
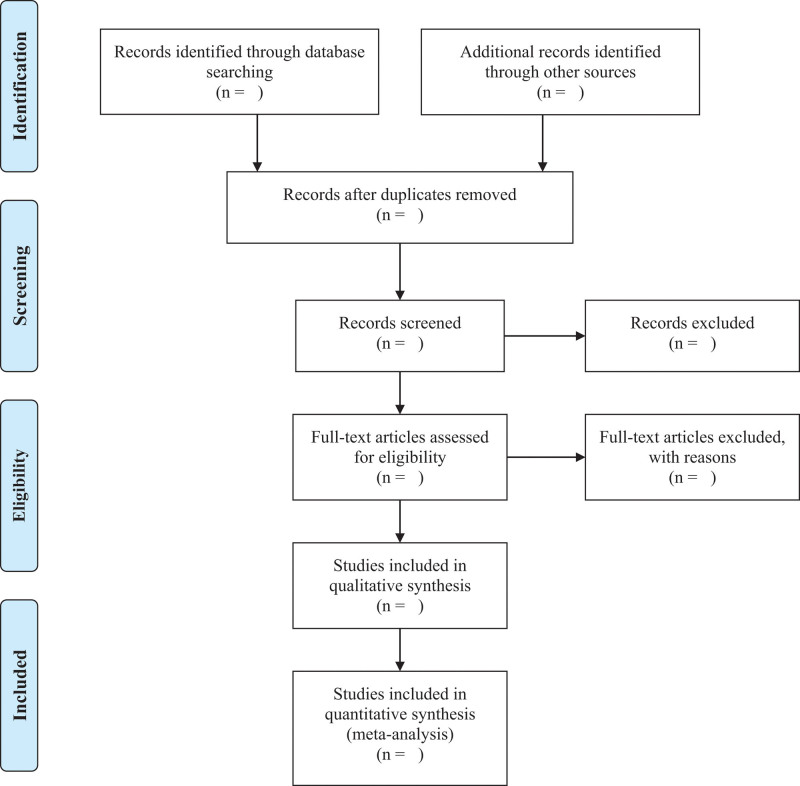
PRISMA flow diagram of study identification and selection.

EndNote X9 (Thomson Reuters, New York, NY) software will be used to manage the literature and perform filtering, and categorize the document and remove duplicates. After classifying the literature and removing duplicates, 2 independent reviewers (WZ and FJX) review the titles and abstracts of the identified studies to exclude irrelevant parts. Then 2 reviewers (WZ and FJX) will read the full text and finally determine the research suitable for network meta-analysis. During this process, if there is any disagreement or inconsistency between the 2 reviewers, ask a third researcher (SJJ) to help make a judgment. Figure 1 is a schematic diagram of literature selection in this study.

### 3.5. Data extraction

Two independent reviewers (FJX and YML) used Microsoft Excel to independently extract and manage the data. The extracted data items include:

1) *Study characteristics*: title, first author name, publication year, funding, random method.
2) *Participants*: sample size, gender, average age, race, diagnostic criteria, disease course.
3) *Intervention*: type of therapy, clinical dosage, course of treatment.
4) *Results*: primary and secondary results, type of outcome measurement, adverse events, and follow-up time.

Before the formal data extraction, 10 studies were randomly selected to test and modify the pre-designed table. In the data extraction process, if there is any objection can be negotiated, or by a third reviewer (SJJ) accuracy and consistency checking of data.

### 3.6. Risk of bias assessment

According to the Cochrane Collaboration Risk of Bias Tool (http://handbook-5-1.cochrane.org/), 2 reviewers (SGF and XHL) independently assessed the risk of bias, which included the 7 specific domains: random sequence generation, allocation concealment, blinding of participants and personnel, blinding of outcome data, incomplete outcome data, selective reporting, and other bias. If necessary, will be the third reviewer (WZ) to solve different opinions.

### 3.7. Data synthesis

We will generate descriptive statistics for the trial, and study population characteristics across all eligible trials, describing the types of comparisons and some important variables, either clinical or methodological (such as year of publication, age, severity of illness, sponsorship and clinical setting). We will draw the network diagram to graphically present the available evidence.

### 3.8. Pairwise meta-analyses

For each direct treatment, more than 2 RCTs should be performed for paired meta-analyses to study the role of oral Chinese patent drugs in treatment by visually inspecting the forest plots. We will use the SMD as the effect measure for continuous outcome variables, and use odds ratio to evaluate the effect size of dichotomous variables. The heterogeneity of this analysis will be assessed by Cochrane χ^2^ test and *I*^2^ statistics, a fixed-effect model will be used to analyze the data if there is no heterogeneity (*P* ≥ .1, *I*^2^ ≤ 50%). If there is evidence of heterogeneity (*P* < .1, *I*^2^ > 50%), a random-effect model will be used, and the possible causes will be searched from both methodological and clinical. In this analysis, we also used the tool described in the Cochrane Collaboration Handbook (http://handbook-5-1.cochrane.org/) as a reference guide for assessing heterogeneity.

### 3.9. Network meta-analysis

Network element can compare multiple interventions at the same time, while retaining the internal randomization of individual trials. Therefore, we will use the network meta-analysis model for all studies within the Bayesian framework. Using a random effects network meta-analysis to analyze the primary and secondary outcomes, we will estimate all possible pairwise relative effects and rank the interventions for each oral Chinese patent medicine. For each outcome, the distribution of ranking probabilities and surface under the cumulative ranking curve are used to estimate the relative rank of different oral Chinese patent medicines.^[40]^ In this study, WinBUGS V.1.4.3.37 and STATA V.13 will be used to analyze the data.^[41]^ Report the results in standardized mean differences, along with 95% confidence interval (CI) based on 100,000 Monte Carlo simulations and vague priors. Model convergence will be assessed by examining trace and history plots and by calculating the Gelman-Rubin statistic. Systematic descriptions will be provided instead if the heterogeneity between studies is too great, especially if the clinical or methodological heterogeneity is too great to perform a meta-analysis.

### 3.10. Assumption of transitivity

We will evaluate the transitivity assumption by comparing the distribution of clinical and methodological variables that are considered as potential effect modifiers (e.g., changes in AMI severity, medication, coronary lumen status after PCI, attack frequency of angina pectoris or duration, mean baseline cardiac function grading and age) across oral Chinese patent medicines interventions.^[42,43]^

### 3.11. Assessment of inconsistency

Inconsistent including inconsistent cycle and design, is the same compare the difference between direct and indirect effect estimates.^[44]^ We will use the node-splitting method to evaluate whether there is inconsistency between direct and indirect evidence for the same comparison,^[45]^ and *P* > .05 indicates consistency. As far as we know, the effect of the inconsistency test is low.^[46]^ Therefore, we need to be careful with inconsistent statistical inferences.

### 3.12. Subgroup and sensitivity analyses

We will explore the possible sources of significant inconsistency or heterogeneity by meta-regression analyses and subgroup. If data are sufficient, subgroup analyses will be performed in terms of age, sex ratio, cardiac function grade before PCI, comorbidities, duration of treatment, and study quality. It is well known that TCM research always involves the problem of syndrome differentiation and treatment.^[47]^ The oral Chinese patent medicines in this study are mainly used to remove blood stasis, supplement *Qi* and nourish *Yin*, regulate *Qi* and expand chest to relieve pain. Therefore, we will perform subgroup analyses on different syndrome types. If more than 10 studies were available, we will perform meta-regression analyses. We will recalculate the summary effect by excluding studies one by one for sensitivity analyses, and observe the impact on the final results.^[48]^

### 3.13. Small-study effects and publication bias

We will use a comparison-adjusted funnel plot to evaluate the comparison and result of each treatment for small-study effects. If the meta-analysis comprises ≥10 trials, a funnel plot will be used to assess publication bias.^[49]^ Then use Egger’s test to evaluate the asymmetry of the funnel plot.

### 3.14. Quality of evidence

Using GRADEprofiler (GRADEpro3.6software, The cochrane Collaboration, Britain), we will use the Grading of Recommendations Assessment, Development and Evaluation guidelines to assess the quality of evidence and strength of recommendations for the main outcomes.^[50]^ The quality of evidence will be divided into 4 levels: very low, low, moderate and high. Study limitations (risk of bias), indirectness, inconsistency, publication bias, and inaccuracies can reduce the quality of evidence, whereas residual confounding, dose-response gradients, and large magnitude of effect can improve the quality of evidence.

## 4. Discussion

Although the primary prevention of coronary heart disease has improved, cardiovascular disease is still the main cause of human morbidity and death, and its prevalence has continued and rapidly increased in the past few decades, causing a huge social and economic burden worldwide.^[51]^ PCI is the main method for the treatment of ST-elevation myocardial infarction in China.^[52]^ However, although PCI can significantly alleviate the symptoms of AMI and reduce the incidence of complications after AMI, studies have shown that the mortality of patients with stent thrombosis within 30 days is about 20% to 45%,^[53]^ indicating that there is still a risk of adverse cardiac events and even death after PCI.^[54]^ The proposed network meta-analysis will provide evidence of the comparative safety and effectiveness of oral Chinese patent medicines for AMI after PCI on the national essential drugs list of China. By the network meta-analysis, we hope to provide clinicians with valuable evidence, while being able to aid decision-making for patients, as well as guide future studies. However, this study also has some limitations. The heterogeneity among studies may be high, which may impact the final results. We will publish the findings and results of this research in a peer-reviewed journal.

## Author contributions

SJJ was responsible for this study. SJJ, WZ, and FJX conceived and designed the study. SGF, FJX, XHL, and YML participated in drafting the protocol and preparing the manuscript. All authors read and approved the final manuscript.

**Conceptualization:** Wei Zhao, Fan-Jie Xiong.

**Data curation:** Wei Zhao, Fan-Jie Xiong, Shu-Gui Feng.

**Methodology:** Wei Zhao, Fan-Jie Xiong, Shu-Gui Feng, Yan-Ming Li, Xing-Hua Lei.

**Writing – original draft:** Wei Zhao, Fan-Jie Xiong, Yan-Ming Li, Xing-Hua Lei.

**Writing – review & editing:** Wei Zhao, Fan-Jie Xiong, Yan-Ming Li, Xing-Hua Lei, Shi-Jian Jia.

## References

[1] Reed GW Rossi JE Cannon CP. Acute myocardial infarction. Lancet. 2017;389:197–210. 27502078 10.1016/S0140-6736(16)30677-8

[2] Benjamin EJ Muntner P Alonso A. Heart disease and stroke statistics-2019 update: a report from the American Heart Association. Circulation. 2019;139:e56–28. 30700139 10.1161/CIR.0000000000000659

[3] Thygesen K Alpert JS Jaffe AS. Joint ESC/ACCF/AHA/WHF Task Force for Universal Definition of Myocardial Infarction. Third universal definition of myocardial infarction. J Am Coll Cardiol. 2012;60:1581–98. 22958960 10.1016/j.jacc.2012.08.001

[4] Richardson PD Davies MJ Born GV. Influence of plaque configuration and stress distribution on fissuring of coronary atherosclerotic plaques. Lancet. 1989;2:941–4. 2571862 10.1016/s0140-6736(89)90953-7

[5] Loree HM Kamm RD Stringfellow RG Lee RT. Effects of fibrous cap thickness on peak circumferential stress in model atherosclerotic vessels. Circ Res. 1992;71:850–8. 1516158 10.1161/01.res.71.4.850

[6] Andreotti F Becker RC. Atherothrombotic disorders: new insights from hematology. Circulation. 2005;111:1855–63. 15824214 10.1161/01.CIR.0000160361.73423.23

[7] Trip MD Cats VM Van Capelle FJ Vreeken J. Platelet hyperreactivity and prognosis in survivors of myocardial infarction. N Engl J Med. 1990;322:1549–54. 2336086 10.1056/NEJM199005313222201

[8] Goldschmidt-Clermont PJ Creager MA Losordo DW. Atherosclerosis 2005: recent discoveries and novel hypotheses. Circulation. 2005;112:3348–53. 16301361 10.1161/CIRCULATIONAHA.105.577460

[9] Clark AM Haykowsky M Kryworuchko J. A meta-analysis of randomized control trials of home-based secondary prevention programs for coronary artery disease. Eur J Cardiovasc Prev Rehabil. 2010;17:261–70. 20560165 10.1097/HJR.0b013e32833090ef

[10] Liew R Sulfi S Ranjadayalan K Cooper J Timmis AD. Declining case fatality rates for acute myocardial infarction in South Asian and white patients in the past 15 years. Heart. 2006;92:1030–4. 16387823 10.1136/hrt.2005.078634 PMC1861115

[11] Roffi M Patrono C Collet JP. ESC Scientific Document Group. 2015 ESC guidelines for the management of acute coronary syndromes in patients presenting without persistent ST-segment elevation: task force for the management of acute coronary syndromes in patients presenting without persistent ST-Segment Elevation of the European Society of Cardiology (ESC). Eur Heart J. 2016;37:267–315. 26320110 10.1093/eurheartj/ehv320

[12] Burkhoff D Mirsky I Suga H. Assessment of systolic and diastolic ventricular properties via pressure-volume analysis: a guide for clinical, translational, and basic researchers. Am J Physiol Heart Circ Physiol. 2005;289:H501–12. 16014610 10.1152/ajpheart.00138.2005

[13] Reyentovich A Barghash MH Hochman JS. Management of refractory cardiogenic shock. Nat Rev Cardiol. 2016;13:481–92. 27356877 10.1038/nrcardio.2016.96

[14] Goto Y Igarashi Y Yamada O Hiramori K Suga H. Hyperkinesis without the Frank-Starling mechanism in a nonischemic region of acutely ischemic excised canine heart. Circulation. 1988;77:468–77. 3338135 10.1161/01.cir.77.2.468

[15] Klapdor B Ewig S Schaberg T. CAPNETZ Study Group. Presentation, etiology and outcome of pneumonia in younger nursing home residents. J Infect. 2012;65:32–8. 22330772 10.1016/j.jinf.2012.02.003

[16] Davies MJ. The pathophysiology of acute coronary syndromes. Heart. 2000;83:361–6. 10677422 10.1136/heart.83.3.361 PMC1729334

[17] Libby P. Mechanisms of acute coronary syndromes and their implications for therapy. N Engl J Med. 2013;368:2004–13. 23697515 10.1056/NEJMra1216063

[18] Badimon L Padró T Vilahur G. Atherosclerosis, platelets and thrombosis in acute ischaemic heart disease. Eur Heart J Acute Cardiovasc Care. 2012;1:60–74. 24062891 10.1177/2048872612441582 PMC3760546

[19] Davì G Patrono C. Platelet activation and atherothrombosis. N Engl J Med. 2007;357:2482–94. 18077812 10.1056/NEJMra071014

[20] Tibaut M Mekis D Petrovic D. Pathophysiology of myocardial infarction and acute management strategies. Cardiovasc Hematol Agents Med Chem. 2017;14:150–9. 27993119 10.2174/1871525714666161216100553

[21] Soar J Nolan JP Böttiger BW. Adult Advanced Life Support Section Collaborators. European resuscitation council guidelines for resuscitation 2015: section 3. adult advanced life support. Resuscitation. 2015;95:100–47. 26477701 10.1016/j.resuscitation.2015.07.016

[22] Nikolaou NI Arntz HR Bellou A Beygui F Bossaert LL Cariou A; Initial Management of Acute Coronary Syndromes Section Collaborator. European resuscitation council guidelines for resuscitation 2015 section 8. Initial management of acute coronary syndromes. Resuscitation. 2015;95:264–77. 26477416 10.1016/j.resuscitation.2015.07.030

[23] Kolh P Windecker S Alfonso F. European Society of Cardiology Committee for Practice Guidelines. 2014 ESC/EACTS guidelines on myocardial revascularization: the task force on myocardial revascularization of the European Society of Cardiology (ESC) and the European Association for Cardio-Thoracic Surgery (EACTS). Developed with the special contribution of the European Association of Percutaneous Cardiovascular Interventions (EAPCI). Eur J Cardiothorac Surg. 2014;46:517–92. 25173601 10.1093/ejcts/ezu366

[24] Bueno H Pocock S Danchin N. International patterns of dual antiplatelet therapy duration after acute coronary syndromes. Heart. 2017;103:132–8. 27504002 10.1136/heartjnl-2016-309509 PMC5284475

[25] Kourlaba G Fragoulakis V Maniadakis N. Economic evaluation of clopidogrel in acute coronary syndrome patients without ST-segment elevation in Greece: a cost-utility analysis. Appl Health Econ Health Policy. 2012;10:261–71. 22667992 10.2165/11633820-000000000-00000

[26] Kloner RA Ganote CE Jennings RB. The “no-reflow” phenomenon after temporary coronary occlusion in the dog. J Clin Invest. 1974;54:1496–508. 4140198 10.1172/JCI107898 PMC301706

[27] Rezkalla SH Kloner RA. Coronary no-reflow phenomenon: from the experimental laboratory to the cardiac catheterization laboratory. Catheter Cardiovasc Interv. 2008;72:950–7. 19021281 10.1002/ccd.21715

[28] Eeckhout E Kern MJ. The coronary no-reflow phenomenon: a review of mechanisms and therapies. Eur Heart J. 2001;22:729–39. 11350105 10.1053/euhj.2000.2172

[29] Tsetskhladze E Khintibidze I. Retrospective study of evaluation of patients with ST elevation myocardial infarction and intact coronary arteries. Evaluation of treatment approaches; outcome and prognosis. Georgian Med News. 2017;(267):48–52. 28726653

[30] Xutian S Cao D Wozniak J Junion J Boisvert J. Comprehension of the unique characteristics of traditional Chinese medicine. Am J Chin Med. 2012;40:231–44. 22419419 10.1142/S0192415X12500188

[31] Guo XY Liu J Liu J. Use of traditional Chinese medicine in Chinese patients with coronary heart disease. Biomed Environ Sci. 2013;26:303–10. 23534471 10.3967/0895-3988.2013.04.009

[32] Hao PP Jiang F Chen YG. Traditional Chinese medication for cardiovascular disease. Nat Rev Cardiol. 2015;12:115–22. 25384847 10.1038/nrcardio.2014.177

[33] National Health Commission of the People’s Republic of China. The National Essential Drugs List of China (2018 edition). Beijing: People’s Medical Publishing House 2018.

[34] Sung HJ Shin HK. Comparision and Research on Policy of Traditional Medicine in Korea and Three Countries of East Asia. Korean Institute of Oriental Medicine 1997. (Korean).

[35] World Health Organization. WHO Traditional Medicine Strategy 2005-2005. World Health Organization 2002. Available at: https://www.who.int/publications/i/item/WHO-EDM-TRM-2002.1.

[36] Li M Li C Chen S. Potential effectiveness of Chinese patent medicine tongxinluo capsule for secondary prevention after acute myocardial infarction: a systematic review and meta-analysis of randomized controlled trials. Front Pharmacol. 2018;9:830. 30123126 10.3389/fphar.2018.00830 PMC6085586

[37] Luo J Xu H Chen K. Systematic review of compound danshen dropping pill: a Chinese patent medicine for acute myocardial infarction. Evid Based Complement Alternat Med. 2013;2013:808076. 23843882 10.1155/2013/808076 PMC3703382

[38] Shamseer L Moher D Clarke M. PRISMA-P Group. Preferred reporting items for systematic review and meta-analysis protocols (PRISMA-P) 2015: elaboration and explanation. BMJ. 2015;350:g7647. 25555855 10.1136/bmj.g7647

[39] Virani SS Alonso A Benjamin EJ. American Heart Association Council on Epidemiology and Prevention Statistics Committee and Stroke Statistics Subcommittee. Heart disease and stroke statistics-2020 update: a report from the American heart association. Circulation. 2020;141:e139–596. 31992061 10.1161/CIR.0000000000000757

[40] Salanti G Ades AE Ioannidis JP. Graphical methods and numerical summaries for presenting results from multiple-treatment meta-analysis: an overview and tutorial. J Clin Epidemiol. 2011;64:163–71. 20688472 10.1016/j.jclinepi.2010.03.016

[41] Crainiceanu CM Goldsmith AJ. Bayesian functional data analysis using WinBUGS. J Stat Softw. 2010;32:i11. 21743798 PMC3130307

[42] Dias S Welton NJ Sutton AJ. NICE DSU Technical Support Document 2: A Generalised Linear Modelling Framework for Pairwise and Network Meta-analysis of Randomised Controlled Trials. London: National Institute for Health and Care Excellence (NICE) 2014. 27466657

[43] Dias S Welton NJ Sutton AJ. NICE DSU Technical Support Document 4: Inconsistency in Networks of Evidence Based on Randomised Controlled Trials. London: National Institute for Health and Care Excellence (NICE) 2014. 27466656

[44] White IR Barrett JK Jackson D Higgins JPT. Consistency and inconsistency in network meta-analysis: model estimation using multivariate meta-regression. Res Synth Methods. 2012;3:111–25. 26062085 10.1002/jrsm.1045 PMC4433771

[45] Dias S Welton NJ Caldwell DM Ades AE. Checking consistency in mixed treatment comparison meta-analysis. Stat Med. 2010;29:932–44. 20213715 10.1002/sim.3767

[46] Veroniki AA Mavridis D Higgins JP. Characteristics of a loop of evidence that affect detection and estimation of inconsistency: a simulation study. BMC Med Res Methodol. 2014;14:106. 25239546 10.1186/1471-2288-14-106 PMC4190337

[47] Jiang M Lu C Zhang C. Syndrome differentiation in modern research of traditional Chinese medicine. J Ethnopharmacol. 2012;140:634–42. 22322251 10.1016/j.jep.2012.01.033

[48] Chiavaroli L Kendall CWC Braunstein CR. Effect of pasta in the context of low-glycaemic index dietary patterns on body weight and markers of adiposity: a systematic review and meta-analysis of randomised controlled trials in adults. BMJ Open. 2018;8:e019438. 10.1136/bmjopen-2017-019438 PMC5884373 29615407

[49] Egger M Davey Smith G Schneider M Minder C. Bias in meta-analysis detected by a simple, graphical test. BMJ. 1997;315:629–34. 9310563 10.1136/bmj.315.7109.629 PMC2127453

[50] Joshi R Alim M Kengne AP. Task shifting for non-communicable disease management in low and middle income countries – a systematic review. PLoS One. 2014;9:e103754. 25121789 10.1371/journal.pone.0103754 PMC4133198

[51] Montalescot G Dallongeville J Van Belle E. OPERA Investigators. STEMI and NSTEMI: are they so different? 1 year outcomes in acute myocardial infarction as defined by the ESC/ACC definition (the OPERA registry). Eur Heart J. 2007;28:1409–17. 17412730 10.1093/eurheartj/ehm031

[52] Liu S Li Y Zeng X. Burden of cardiovascular diseases in China, 1990-2016: findings from the 2016 global burden of disease study. JAMA Cardiol. 2019;4:342–52. 30865215 10.1001/jamacardio.2019.0295 PMC6484795

[53] Reejhsinghani R Lotfi AS. Prevention of stent thrombosis: challenges and solutions. Vasc Health Risk Manag. 2015;11:93–106. 25657588 10.2147/VHRM.S43357 PMC4315466

[54] Thiele H Akin I Sandri M. CULPRIT-SHOCK Investigators. PCI strategies in patients with acute myocardial infarction and cardiogenic shock. N Engl J Med. 2017;377:2419–32. 29083953 10.1056/NEJMoa1710261

